# A Comprehensive Review of Artificial Intelligence in Prevention and Treatment of COVID-19 Pandemic

**DOI:** 10.3389/fgene.2022.845305

**Published:** 2022-04-26

**Authors:** Haishuai Wang, Shangru Jia, Zhao Li, Yucong Duan, Guangyu Tao, Ziping Zhao

**Affiliations:** ^1^ College of Computer Science, Zhejiang University, Hangzhou, China; ^2^ Department of Computer and Information Engineering, Tianjin Normal University, Tianjin, China; ^3^ Alibaba-ZJU Joint Research Institute of Frontier Technologies, Zhejiang University, Hangzhou, China; ^4^ College of Computer Science and Technology, Hainan University, Haikou, China; ^5^ Department of Radiology, Shanghai Chest Hospital, Shanghai Jiaotong University, Shanghai, China

**Keywords:** Artificial Intelligence, clinical diagnosis, COVID-19, medical imaging, Pandemic Prediction, pandemic, COVID-19 review, telemedicine

## Abstract

The unprecedented outbreak of the Corona Virus Disease 2019 (COVID-19) pandemic has seriously affected numerous countries in the world from various aspects such as education, economy, social security, public health, etc. Most governments have made great efforts to control the spread of COVID-19, e.g., locking down hard-hit cities and advocating masks for the population. However, some countries and regions have relatively poor medical conditions in terms of insufficient medical equipment, hospital capacity overload, personnel shortage, and other problems, resulting in the large-scale spread of the epidemic. With the unique advantages of Artificial Intelligence (AI), it plays an extremely important role in medical imaging, clinical data, drug development, epidemic prediction, and telemedicine. Therefore, AI is a powerful tool that can help humans solve complex problems, especially in the fight against COVID-19. This study aims to analyze past research results and interpret the role of Artificial Intelligence in the prevention and treatment of COVID-19 from five aspects. In this paper, we also discuss the future development directions in different fields and prove the validity of the models through experiments, which will help researchers develop more efficient models to control the spread of COVID-19.

## Introduction

**FIGURE 1:**
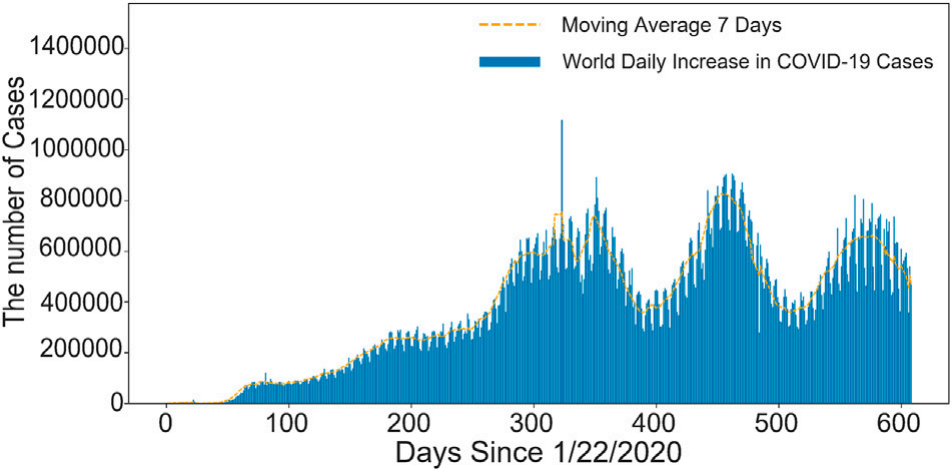
The number of new confirmed cases worldwide every day. The abscissa is the timeline and the ordinate is the number of COVID-19 confirmed cases. The number of COVID-19 cases is increasing in the first 300 days, and there is a wavy line in the second 300 days.

**FIGURE 2:**
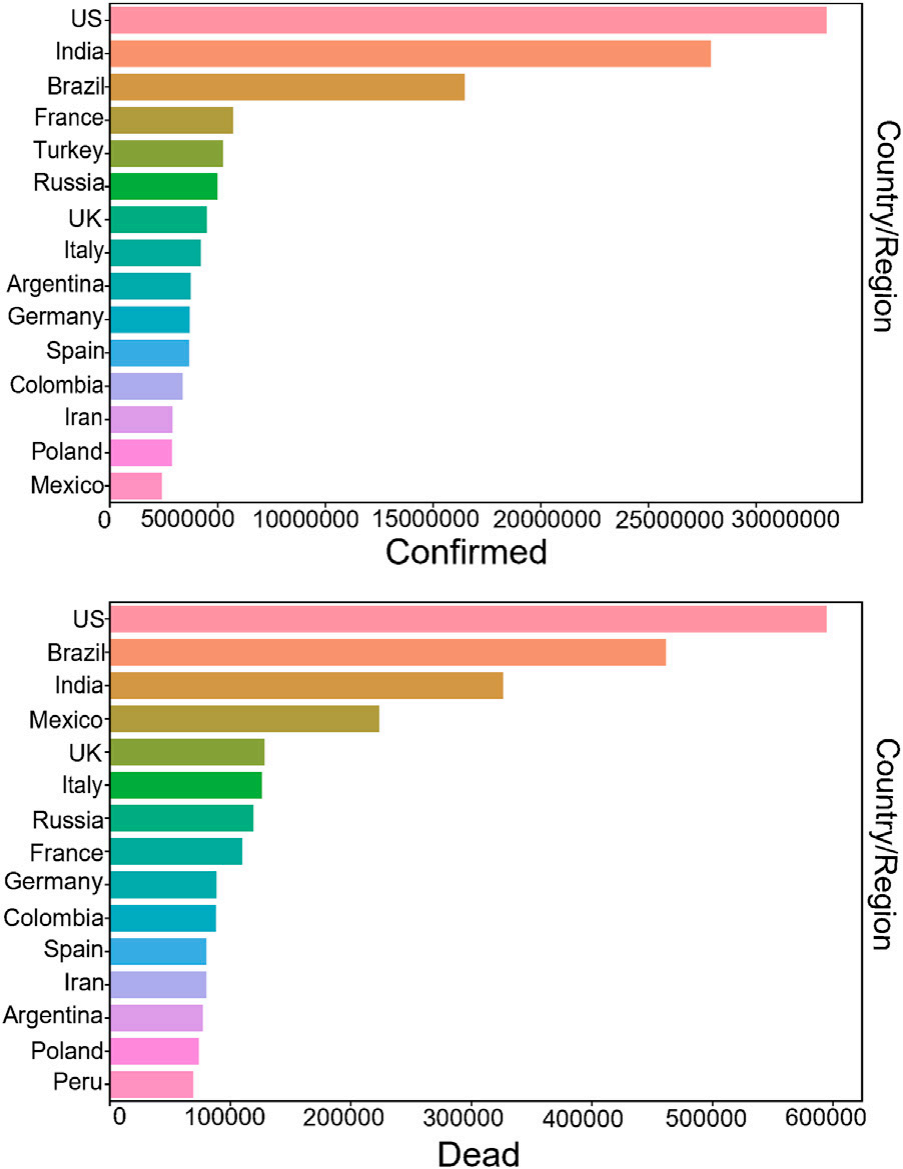
Top 15 countries with cumulative confirmed cases and deaths. Confirmed cases are shown above, and deaths are shown below. The United States, India and Brazil are the top three, with the United States having the most cumulative confirmed cases and deaths.

In December 2019, COVID-19 hit Hubei, China, and many pneumonia cases of unknown cause were found in some hospitals in Wuhan. The pandemic has been infecting millions of people afterwards, which was eventually confirmed as an acute respiratory infection caused by Novel Coronavirus 2019 infection. On 11 February 2020, the World Health Organization (WHO) named it “COVID-19” (Wang et al., 2020a; He et al., 2020; Sohrabi et al., 2020), and the fight against COVID-19 began around the world. This disease is a highly contagious and highly pathogenic infectious disease, which may cause various forms of disease from mild to severe (Chen et al., 2020a; Paules et al., 2020). For example, it can transfer the mild self-limiting respiratory illness to severe pneumonia and even cause multiple organ failure, or death. Up till to 23 September 2021, there have been 230,773,965 COVID-19 infections worldwide, as shown in Figure 1, the number of confirmed COVID-19 infections is still increasing. Figure 2 shows the top 15 countries with the highest cumulative number of confirmed cases and the highest number of deaths globally, where the top three are United States, Brazil and India. [The data in Figures 1, 2 are from the website: https://github.com/CSSEGISandData/COVID-19. This COVID-19 data repository is from the Center for Systems Science and Engineering (CSSE) at Johns Hopkins University. The data was downloaded on 20 September 2021]. Thus, it is worth thinking about what caused the pandemic. The outbreak of the pandemic is due to the lack of relevant information in the early stages and the prediction of its future transmission, resulting in delayed national containment measures and low awareness of self-protection among the population. Moreover, in some areas with poor medical conditions, there is not enough vaccine for the public, and patients cannot afford systematic treatment or expensive hospital expenses, thereby they have to self-isolate which greatly increased the risk of infection.

At present, due to the lack of effective antiviral drugs for COVID-19, patients with mild symptoms can be treated with general treatment, such as bed rest, timely and effective oxygen therapy, appropriate application of antibiotics, antiviral therapy and glucocorticoid therapy, etc. In the treatment of critically ill patients, the treatment principle is based on general treatment, such as actively prevent complications, treat basic diseases, prevent secondary infections, support organ functions, and respiratory support, etc. However, these methods are not able to completely stop the death toll from rising, hence, developing a drug that targets COVID-19 would be an effective way to stop the spread of the pandemic (Bayat et al., 2021).

Recently, more and more AI researchers have devoted to the prevention and treatment of COVID-19 from different fields (Chamola et al., 2020), including clinical medicine, economics, infectious diseases, computer science, psychology, government management, etc. Therefore, Artificial Intelligence is able to help us better understand the protein structure of COVID-19 virus and develop effective drugs to cure patients (Rahman et al., 2020; Soomro et al., 2022), which will greatly save the time of drug design and vaccine development. It can also diagnose whether it is infected by learning clinical data and Computed Tomography (CT) images, which greatly saves the problem of manpower shortage, in order to help to control suspected patients as soon as possible, and implement measures such as isolation and monitoring (Yu et al., 2020). Second, machine learning can also be used to make reasonable predictions about the future development trend of the COVID-19, so as to help decision-makers implement corresponding control measures to prevent the spread of COVID-19. Finally, the construction of telemedicine platform is inseparable from the participation of AI. Therefore, AI plays an extremely important role in combating the COVID-19 pandemic.

Nowadays, researchers have been widely applying AI to against the outbreak of COVID-19. In this paper, we aim to systematically review the active role of AI in prevention the outbreak of COVID-19 pandemic, and the current challenges in the related research. In addition, we also summarized and demonstrated the recently studies in terms of the results and conclusions from different aspects. Chapter 2 discusses the interpretation of medical images by AI. Chapter 3 introduces the use of clinical data modeling to detect the severity of patients. Chapter 4 discusses the application of AI in the treatment of patients with COVID-19. Chapter 5 summarizes the COVID-19 epidemic prediction model represented by mathematical models and machine learning models. Chapter 6 introduces the current development of telemedicine technology. Finally, the challenges and future development directions of AI technology in the prevention and treatment of COVID-19 are discussed in Chapter 7.

## Artificial Intelligence Interpretation of Chest Radiology Images

Recently, with the development of computer technology, AI interpretation of medical images can help doctors identify and detect the types of diseases and determine the affected areas. As COVID-19 is persistently ravaging the world, researchers have been leveraging medical images (e.g., chest X-rays and CT images) as the main tools for COVID-19 diagnosis. This section summarizes the main methods of medical imaging for COVID-19 in Table 1. Methods based on deep learning, such as deep feature extraction, pre-trained Convolutional Neural Network (CNN) and end to end training CNN models, have been widely used for image classification tasks. For depth feature extraction, most of the preprocessed depth CNN models are used, such as Residual Neural Network 18 (ResNet18), Residual Neural Network 50 (ResNet50), Residual Neural Network 101 (ResNet101), Visual Geometry Group 16 (VGG16) and Visual Geometry Group 19 (VGG19). For the classification of deep features, a Support Vector Machine (SVM) classifier is used together with various functions, e.g., Linear, Quadratic, Cubic and Gaussian, etc.

**TABLE 1:** Main methods of Medical Imaging for COVID-19.

Classifier	Data set	Accuracy	Data availability	References
CNN	2000 x-rays images (162 COVID-19 positive, 4280 common pneumonia positive, 400 TB positive)	99.92%	https://github.com/ieee8023/covid-chestxray-dataset	Das et al. (2020)
CNN + PCA	500 X-ray images (250 COVID-19 positive cases and 250 normal healthy cases.)	97.6–100%	https://www.kaggle.com/paultimothymooney/chest-xray-pneumonia	Rasheed et al. (2021)
CNN + ACGAN	1124 X-ray images (403 images of COVID-19 and 721 normal images)	95%	https://github.com/agchung/Figure1-COVID-chestxray-dataset	Waheed et al. (2020)
Based on deep convolutional neural network CovXNet	1583 normal X-ray images, (1493 COVID-19 pneumonia X-ray images and 2780 bacterial pneumonia X-ray images)	97.4% (Second category)	https://github.com/Perceptron21/CovXNet	Mahmud et al. (2020)
		90.2% (Multiple categories)		
Deep CNN transfer learning method	423 COVID-19, 1485 viral pneumonia and 1579 normal chest X-ray images	99.7% (Second category)	https://www.kaggle.com/tawsifurrahman/covid19-radiography-database	Chowdhury et al. (2020)
		97.9% (Three categories)		
Deep CNN model CoroNet	X-ray images of 1203 normal cases, 1591 viral pneumonia cases	95% (Three categories)	https://github.com/drkhan107/CoroNet	Khan et al. (2020)
		93% (Four categories)		
COVID-Net	COVID X Open access to the benchmark data set (13,975 CXR images, 358 COVID-19 CXR images.)	98.9%	https://github.com/lindawangg/COVID-Net	Wang et al. (2020b)
nCOVnet	142 COVID-19 X-ray images 5863 non-COVID-19 X-ray images	97%	https://github.com/ieee8023/covid-chestxray-dataset	Panwar et al. (2020)
DenseNet121	2724 C T images (1029 COVID-19 images,)	90.8%	https://wiki.cancerimagingarchive.net/display/Public/LIDC-IDRI	Harmon et al. (2020)
DarkNet model based on Deep Learning	500 normal and 500 COVID-19 images	98.08% (Second category)	https://github.com/muhammedtalo/COVID-19	Tulin et al. (2020)
		87.02% (Multiple categories)		
Deep transfer learning (DTL) model with DenseNet201	1,262 COVID-19 positive images, 1,230 negative images	99.82%	https://www.kaggle.com/plameneduardo/sarscov2-ctscan-dataset	Vijay et al. (2020)
An automated COVID-19 screening (ACoS)	696 normal, 696 pneumonia and 696 COVID-19 X-ray images	98.062%	https://github.com/ieee8023/covid-chestxray-dataset	Chandra et al. (2021)
Based on deep Bayes-Extrusion Network-COVID Diagnosis-Net	X-ray images (1583 normal persons, 4290 cases of common pneumonia, and 76 cases of COVID-19 infection)	100% (Second category)	https://data.mendeley.com/datasets/rscbjbr9sj/2	Ucar and Korkmaz, (2020)
		98.3% (Three categories)		
Deep learning model and transfer learning based on VGG-16	250 COVID-19 images, 2753 other lung diseases images, and 3520 health images	98%	https://github.com/muhammedtalo/COVID-19	Brunese et al. (2020)
A weakly supervised deep learning framework	TCIA Open data set (150 3D volumetric chest CT exams of COVID-19, CAP and NP patients)	92.3%	https://www.cancerimagingarchive.net/collections/	Hu et al. (2020)
A technique based on a deep residual network	1345 viral pneumonia cases, 10,200 normal cases and 3616 COVID-19 cases	92.1% (Four categories)	https://github.com/pawelparker/DNN-lung-infection-Pattern	Panahi et al. (2022)
Transfer learning 29 different types of AI-based models	352 chest X-ray images (51 COVID-19, 21 non-COVID-19,160 pneumonia,54 TB, and 66 normal images)	93.8% (Validation accuracy)	https://github.com/arunsharma8osdd/covidpred	Sharma et al. (2020)
A multi-view feature learning method	1092 X-ray images (364 COVID-19, 364 normal, and 364 pneumonia)	99.82% (Three categories)	https://www.kaggle.com/paultimothymooney/chest-xray-pneumonia	Hamidreza, (2022)

Das et al. (Das et al., 2020) proposed a CNN-based model to identify infected cases from viral pneumonia or healthy cases. This work used 6 datasets which contain 7,000 X-ray images, in order to classify the COVID-19 positive, positive ordinary pneumonia, *tuberculosis* positive and healthy patients. The classification accuracy (AUC) of the model for COVID-19 positive and negative cases was 99.96% (AUC was 1.0). Similarly, it has an accuracy of 99.92% (AUC 0.99) in classifying pneumonia, *tuberculosis* and COVID-19 positive cases. Rasheed et al. (Rasheed et al., 2021) added a dimension reduction method based on Principal Component Analysis (PCA) on the basis of the CNN to further accelerate the learning process and improve classification accuracy by selecting features with high discriminability. The results showed that the overall accuracy was 95.2%–97.6% without PCA and 97.6–100% with PCA for positive case identification. The applicability of PCA dimension reduction is illustrated. In addition, Waheed et al. (Waheed et al., 2020) proposed a method for synthesizing chest X-ray (CXR) images by developing models based on auxiliary classifier Generative Adversarial Networks (GAN), which improved accuracy 10% by adding synthetic images generated by Covid-GAN. Recently, transfer learning has been widely used in this field. Chowdhury et al. (Chowdhury et al., 2020) proposed a robust technique for automatic detection of COVID-19 pneumonia from chest X-ray images by leveraging pre-trained deep learning algorithms to maximize detection accuracy, which achieved 99.7% accuracy.

**FIGURE 3:**
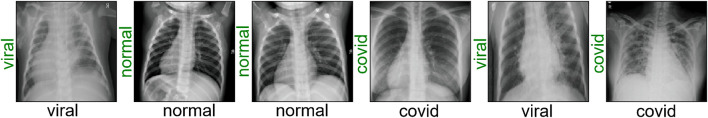
Classification results of Resnet model. If the classification is correct, a green label will appear, otherwise a red label will appear. The Resnet model can correctly classify normal, viral pneumonia, and COVID-19 after training.

**FIGURE 4:**
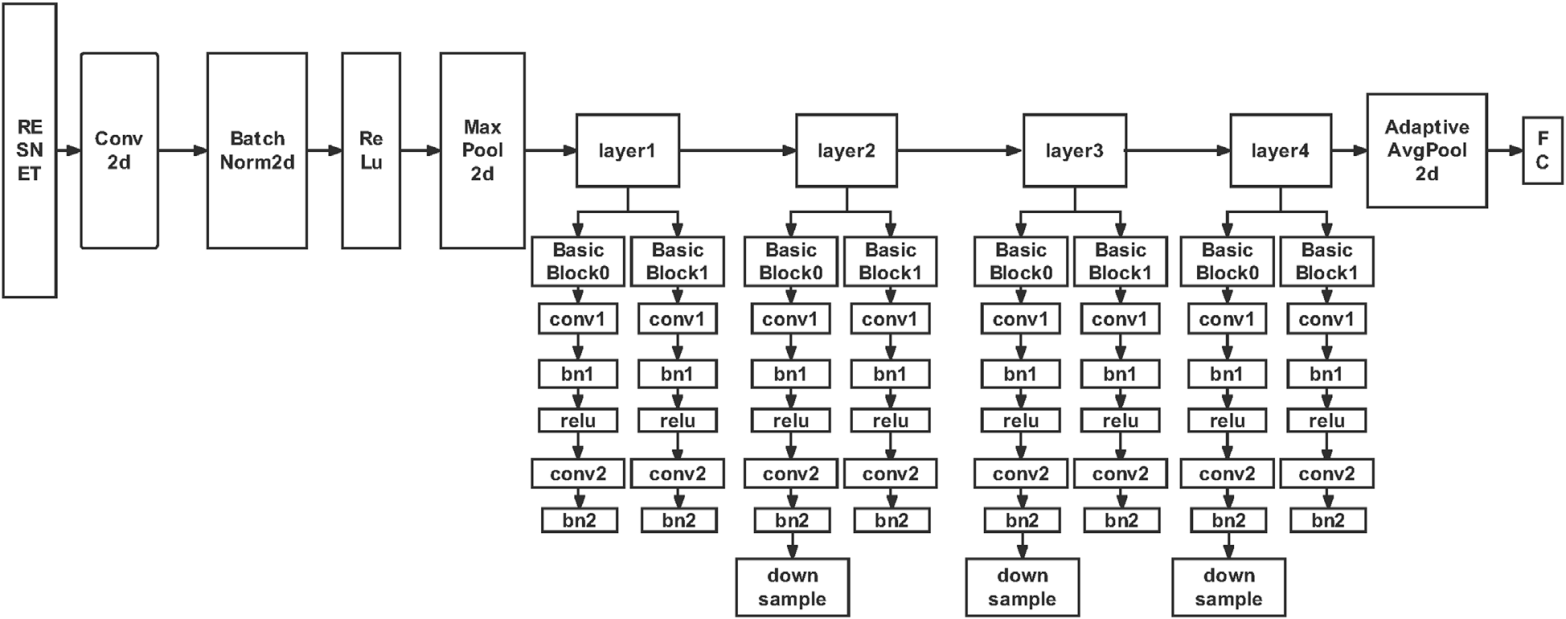
Resnet model framework structure. The characteristic of Resnet is that it is easy to optimize and can improve accuracy by increasing depth. The internal residual block uses jump connection to alleviate the problem of gradient disappearance.

So which model is more effective at detecting COVID-19? Elasnaoui et al. (El Asnaoui and Chawki, 2021) introduced a deep learning model (VGG16, VGG19, Densenet201, Inception_ResNet_V2, Inception_V3, Resnet50, And MobileNet_V2) conducted a comparative study on the detection and classification of COVID-19. Results showed that the use of ResnetV2 and Densnet201 had better results than other models used in this study (accuracy of ResnetV2 and Densnet201 was 92.18 and 88.09%, respectively). Ismael et al. (Ismael and Şengür, 2021) proposed a new end-to-end training CNN model. The Support Vector Machines (SVM) classifier was used to classify the deep features, and different functions were matched. The results show that the deep features extracted from the ResNet50 model and the SVM classifier with linear kernel function produce an accuracy of 94.7%, which is the highest among all the obtained results. In addition, it also shows that deep learning methods are better than local descriptors. Especially the performance of deep features and SVM classifier is better than other methods. In deep feature classification, the cubic function is usually better than all other functions. The ResNet50 model usually produces better results than other preprocessed CNN models. Finally, for end-to-end training, deep CNN models produce better results than shallow networks. Therefore, we also tried to use Resnet to classify chest X-ray images into three categories: normal, viral pneumonia and COVID-19. The accuracy rate in the validation set is 96%. Figure 3 shows the good performance of the model, which can accurately classify X-ray images. The specific structure of this model is shown in Figure 4.

While AI has made some progress in medical imaging, with many models achieving near 100% accuracy on open data sets, there is still a long way to go. We believe that the following points need to be paid attention to in the future: 1. A large open data set is very necessary. So we must continue to increase data sharing and jointly build a complete large-scale database for researchers to use. 2. Hospital imaging data may be incomplete, so we need to improve the accuracy of segmentation and classification to prevent diagnostic errors for COVID-19. 3. As the current epidemic is normalized, we need to develop a system to reduce the pressure on doctors and better apply it to clinical practice. 4. Marking data manually is expensive and time-consuming, so unsupervised deep learning models will be the focus of future research. Finally, we hope that medical image recognition can be deployed to hospitals as soon as possible, so that more patients can receive immediate treatment and save more lives.

## Artificial Intelligence Analysis of Patient Clinical Data

Since 2019, the COVID-19 has gripped the world. The COVID-19 is shockingly transmissible and is constantly mutating. even in an era dominated by information technology, clinical information data on COVID-19 patients is still scarce, and clinical predictions of morbidity, mortality, severity and prognosis are lagging behind. This requires the sharing of Electronic Health Records (EHR) clinical data with researchers and public health agencies. Brat et al. (Brat et al., 2020) formed an International Consortium (4CE) consisting of 96 hospitals in five countries. Successfully leveraged the open source Informatics for Integrating Biology & the Bedside (I2B2) tool KIT10-17 to manage, complete, and share data extracted from the EHR. The goal is to integrate, share and interpret data about the clinical trajectory of patients. Of course, we also hope that more websites around the world can share data with hospitals, which will make a great contribution to clinical intelligence in the future.

As shown in Table 2, currently many researchers have begun to make use of limited clinical data to predict the severity of COVID-19 patients and conduct feature screening for critical indicators in clinical data in combination with Artificial Intelligence methods. Razavian et al. (Razavian et al., 2020) used clinical data and EHR data of 3345 retrospective and 474 prospective hospitalized cases, and based on real-time data values, vital signs and oxygen support variables, established and verified a black box model to identify patients with good prognosis within 96 h. The results showed that the Light Gradient Boosting Machine (Light GBM) model performed well in EHR data, with a positive predictive value of 93.3%. In addition, Arjun et al. (Yadaw et al., 2020) applied machine learning technology to 3841 patients treated by Mount Sinai Health System in New York City, The United States, implemented a systematic machine-learning-based framework by using missing value interpolation, 6 feature selection, 7 classification and 4 statistical techniques. It was found that three highly accessible clinical parameters of patient age, minimum oxygen saturation, and type of patient encounter were fed into an automated Extreme Gradient Boosting (XGBoost) algorithm that accurately classified patients as likely to survive or die. In addition, Liang et al. introduced a machine learning variable selection algorithm called Least Absolute Shrinkage and Selection Operator (LASSO), it was used to identify 10 variables with statistical significance (*p* < 0.05) hazard ratio characteristics (Liang et al., 2020a), (Liang et al., 2020b), a COVID-Gram-based online calculator was developed to allow clinicians to enter the values of the 10 variables required for the risk score and automatically calculate the likelihood of a COVID-19 inpatient developing critical illness (95%CI). Covino et al. used Multivariate proportional hazards (COX) regression to determine the risk factors related to progression (Marcello et al., 2020), (Ji et al., 2020), and a new predictive scoring model was established. Liang also compared the deep learning survival COX model with the classical COX model (Liang et al., 2020b), and found that the deep learning survival COX model is better.

**TABLE 2:** Modeling method of EHR data.

Model	Data set	Result	Important features	Availability	References
Three models, clinical feature model (C model), radiological semantic feature model (R model), and clinical and radiological semantic feature combination model (CR model)	CT images and clinical data from 70 COVID-19 and 66 non-COVID-19 pneumonia patients	The CR model has the highest accuracy and specificity with a maximum AUC of 0.98	GGO with consolidation, tree-in-bud, offending vessel augmentation in lesions, temperature, heart ratio, etc.	https://doi.org/10.1007/s00330-020-06829-2	Chen et al. (2020b)
Four models (Logistic Regression, Random Forest, Light-GBM, and a collection of these three models)	Clinical data and EHR data of 3345 retrospective and 474 prospective Inpatients	The Light-GBM model achieved the best performance on the validation set	Age, Sex, Race, Neutrophils Percent, Lymphocytes Percent, Eosinophils Percent, C-Reactive Protein, C-Reactive Protein, etc.	https://doi.org/10.1038/ s41746-020-00343-x	Razavian et al. (2020)
Recursive Feature Elimination method, Logistic Regression, Support Vector Machine, Random Forest and Extreme Gradient Enhancement (XGBoost) algorithm for prediction.	In 3841 patients at Mount Sinai Health System, 961 retrospective and 249 prospective patients	XGBoost algorithm can accurately classify patients as likely to live or die.	Age, minimum oxygen saturation, and type of patient encounter, etc.	https://github.com/SBCNY/Clinical-predictors-of-COVID-19-mortality	Yadaw et al. (2020)
χ2 test or Kruskal-Wallis test, Multivariate Regression analysis	1,951 charts of confirmed cases in 26 hospitals in Italy.	mortality is predicted by age and the presence of comorbidities.	Age, diabetes, chronic obstructive pulmonary disease (COPD) and chronic kidney disease, etc.	https://www.clinicaltrials.gov	Wang et al. (2020c)
Mann-whitney U, χ2 test, Univariate Cox Analysis	Clinical data from 69 patients	The risk of death in elderly patients may be independent of age, and the presence of severe dementia is a risk factor for this population.	Lactate dehydrogenase and blood oxygen saturation, etc.	https://doi.org/10.1111/ ggi.13960	Marcello et al. (2020)
Minimum absolute contraction selection operator (LASSO) and Logistic Regression	COVID-19 patients from 575 hospitals in 31 provincial-level regions in China	The predictive variables were extracted and the severity of the patients was calculated successfully	Age, Dyspnea, Cancer history, COPD, Comorbidity, X-ray abnormality, etc.	https://github.com/cojocchen/covid19_critically_ill	Liang et al. (2020b)
Multivariate COX Regression	Clinical data of 208 patients	The CALL scoring model was established, and the area under ROC curve was 0.91	Age, Comorbidity, Lymphocyte, D-dimer, LDH, Lymphocyte, etc.	https://doi.org/ 10.1093/cid/ciaa414	Ji et al. (2020)
Machine learning variable selection algorithm for Minimum Absolute Contraction and Selection Operator (LASSO), Combined with Cox deep learning model	1590 patients at 575 medical centers	Deep learning survival Cox model is better than traditional Cox model	Age, hemoptysis, dyspnea, unconsciousness, number of comorbidities, cancer history, neutrophil-to-lymphocyte ratio, etc.	http://118.126.104.170/	Liang et al. (2020a)
Models Based on Whole Clinical Parameters	A publicly available dataset consisting of clinical parameters and protein profile data	The best classification model based on clinical parameters achieved a maximum accuracy of 89.47%	Serum creatinine, age, absolute lymphocyte count, and D-dimer and proteins.	http://14.139.62.220/covidprognosis/	Sardar et al. (2021)
Unsupervised hierarchical clustering and principal component analysis.	Patients. Rotterdam cohort samples	An immune-type based scheme to stratify COVID-19 patients at hospital admittance into high and low risk clinical categories	Serum pro-inflammatory, anti-inflammatory and anti-viral cytokine and anti-SARS-CoV-2 antibody measurements	https://bitbucket.org/immunology-emc/covid_severity_publication/src/master/	Mueller et al. (2022)

Finally, multimodal clinical data information can more accurately diagnose and predict the risk level of patients. Chen et al. (Chen et al., 2020b) combined modeling of medical images and clinical data, and found that the combined model of clinical and radiological semantic features achieved the best effect, with the highest accuracy and specificity, and the maximum AUC was 0.986. Liang (Liang et al., 2020b) added the abnormality of X-ray image into clinical information, and found that the abnormality of X-ray image was the first predictive variable of critical condition. So we hope that future EHR data will be more readily available, a more authoritative and comprehensive database will be established. In this way, our research will be in-depth, and the proposed model is applicable.

**FIGURE 5:**
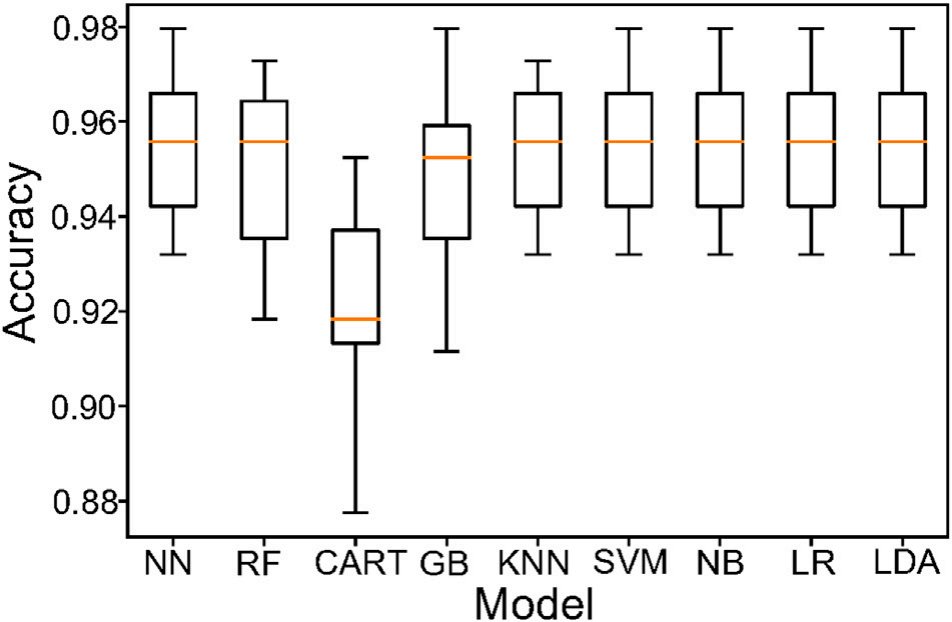
Model comparison of EHR data. Using a box plot to reflect the classification characteristics of different models. The NN,RF,SVM, NB, LR and LDA are better than other models.

At last, we use a variety of EHR data modeling, and it is found that The Neural Network Classifier (NN), Random Forest (RF), K-Nearest Neighbor (KNN), SVM, Naive Bayes (NB), Logistic Regression (LR) and Linear Discriminant Analysis (LDA) have a good effect with an accuracy of 95.4422%. The comparison of different algorithms is shown in Figure 5. We compare the advantages and disadvantages of different algorithms by using a box plot, which is composed of five numerical points, namely, minimum observed value, 25% quantile, median, 75% quantile and maximum observed value. We can conclude from the figure that the average value (yellow line) of NN, RF, KNN, SVM, NB, LR, and LDA is 95.4422%, which is better than Gaussian Bayes (GB) Classification and Regression Trees (CART). Although, we can use the above model to analyze the clinical data of patients, so as to obtain the corresponding prediction results (whether they have COVID-19 or not). Since machine learning model and deep learning model are black box models, we need to study their interpretability more, so that we can understand the mechanism of model prediction and its practicability more easily. In addition, we can combine clinical data with medical imaging data to build a comprehensive model. The problems are analyzed from the multi-modal perspective by integrating various disciplines.

## Artificial Intelligence Discovery of Disease Treatments

At present, COVID-19 has spread all over the world. The cunning virus is constantly mutating and posing a serious threat to human health in the world. It means the use of Artificial Intelligence to identify the host protein and the possible targeting mechanism of the COVID-19 protein has important implications for prevention and treatment of COVID-19. Das et al. (Das et al., 2021) proposed a computational scheme for reconstructing the host virus protein-protein interaction network, using host proteins from 17 important signaling pathways to investigate possible targeting mechanisms of Severe Acute respiratory Syndrome Coronavirus 2 (SARS-CoV-2) proteins. The results showed that Non-structural Proteins (NSP3) and Structural Protein (Spike) were the most influential proteins in interacting with multiple host proteins. The Mitogen-activated Protein Kinase (MAPK) pathway is the most severely affected pathway in SARS-COV-2 infection. Some proteins involved in multiple pathways are highly concentrated in host Protein-Protein Interactions (PPI) and are mainly targeted by multiple viral proteins. The most prominent drug molecules highlighted in the study are arsenic trioxide, dexamethasone and hydroxychloroquine, which may play an important role in preventing deaths. Yaar et al. (Yaar et al., 2021) used Deep Learning (DL), Random Forest (RF), and Gradient Boosted Trees (GBTs) were used to predict the relationship between disease severity and protein in 93 samples (60 COVID-19 patients, 33 controls) and 370 variables from open websites. The study identified TGB1BP2 in cardiovascular group II and MILR1 in inflammatory group as the two most important proteins associated with disease severity. Compared with other algorithms, the proposed model (GBTs) achieves the best prediction of disease severity based on protein. The results also suggest that changes in blood protein associated with the severity of COVID-19 can be used for disease surveillance, early diagnosis and treatment.

In addition, Artificial Intelligence can also be used to discover effective drugs to treat COVID-19. Kong et al. (Kong et al., 2020) described a Web server that can predict binding patterns between COVID-19 targets and ligands, including small molecules, peptides and antibodies. The server provides a friendly interface and binding pattern visualization for the results, which makes it a useful tool for discovering COVID-19 drugs. Wang et al. (Wang, 2020) effectively provided possible treatment options for the outbreak of COVID-19 infectious diseases through computer-aided drug design. This study found that some drugs can act as inhibitors of the major proteases in novel coronavirus, including Carfilzomib, lopinavir et al. Contribute to rational drug design for COVID-19 major proteases. Beck et al. (Beck et al., 2020) used a pretrained Deep-Learning-based drug targeting interaction model, namely molecular converter-drug targeting interaction (MT-DTI), to identify commercially available drugs that act on SARS-COV-2 virus proteins. An antiretroviral drug used for the treatment and prevention of Human Immunodeficiency Virus (HIV) was found to be the best compound, with an inhibitory effect of 94.94 nm against SARS-CoV-2 3C-like proteases. Ton et al. (Ton et al., 2020) introduced a new Deep Learning platform, Deep Docking (DD). The DD combined with Glide can be used to quickly estimate the docking fraction between 1.3 billion chemical structures and the new SARS-CoV-2MPro active site, so that drugs with higher docking fraction can be found compared with known protease inhibitors. Beata et al. (Beata et al., 2020) used Cryo-electron tomography and molecular dynamics simulation were used to help us understand SARS-CoV-2 infection and develop safe vaccines.

At last. Senior et al. (Senior et al., 2020) trained a Neural Network to accurately predict the distance between residue pairs, which conveyed more information about the structure than contact prediction, and determined the most likely three-dimensional shape of the protein through energy minimization. This adds to our understanding of the COVID-19 and helps us develop effective treatments for patients with COVID-19. Schaarschmidt et al. (Schaarschmidt et al., 2017) analyzed protein prediction using Coevolution and Machine Learning methods, compared it with previous CASP experiments, and discussed the results of structure prediction and prediction provided on finite target sets. They found that in more than half of the targets, especially those with many homologous sequences, the accuracy was more than 90%, and in some cases the best predictors were 100% accurate. In conclusion, AI can help us get out of the COVID-19 sooner or later!

**FIGURE 6:**
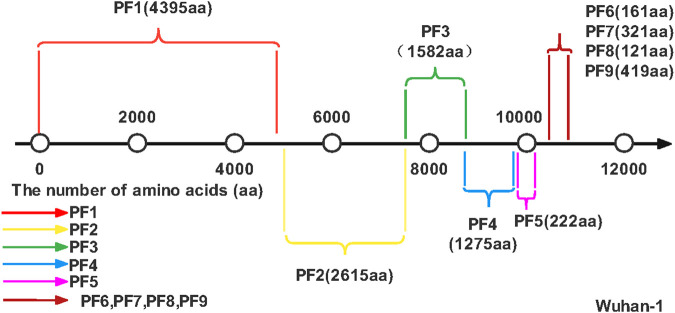
SARS-COV-2 genome. The number line represents the number of amino acids, and different colors represent different Protein Fragments.

Figure 6 shows the genome organization of SARS-COV-2. The organization of a genome is the linear sequence of genetic material (DNA/RNA) and its division into specific functional segments. We can use Artificial Intelligence to extract some Protein Fragment. And compared with SARS-COV genome tissue. In experiments, Although the SARS-COV genome is very similar to that of SARS-COV-2, we know that the DNA/RNA of the two viruses are very different by measuring the editing distance. Finally, The Protein Fragments (PF) such as PF1, PF2, etc. were extracted and the amino acid (aa) numbers of bases on different fragments was obtained. As shown in Figure 6, different colors represent different PF, each PF contains a different number of amino acids, for example, the red line is the first Protein Fragment (PF1), which consists of 4,395 amino acids. Using Artificial Intelligence to study the COVID-19 genome will help enhance our understanding of the virus’s genes and speed up the development of specific drugs and vaccines against COVID-19. In addition, we can also carry out different experiments to screen different drugs, the effect of clinical treatment, and get the best treatment drugs and methods.

## Artificial Intelligence Predictions of COVID-19 Pandemic

As we all know, the rapid spread of the COVID-19 has brought public health departments in some countries to the brink of collapse, with shortages of basic medical equipment such as Intensive Care Unit (ICU) beds, ventilators, masks and protective suits. Therefore, a reasonable AI prediction model plays an important role in predicting the future development trend of COVID-19, formulating scientific and reasonable prevention and control measures, consolidating the existing epidemic prevention achievements, maintaining the safety of life and property of the public and stabilizing the social development order (Foppa et al., 2017).

**FIGURE 7:**
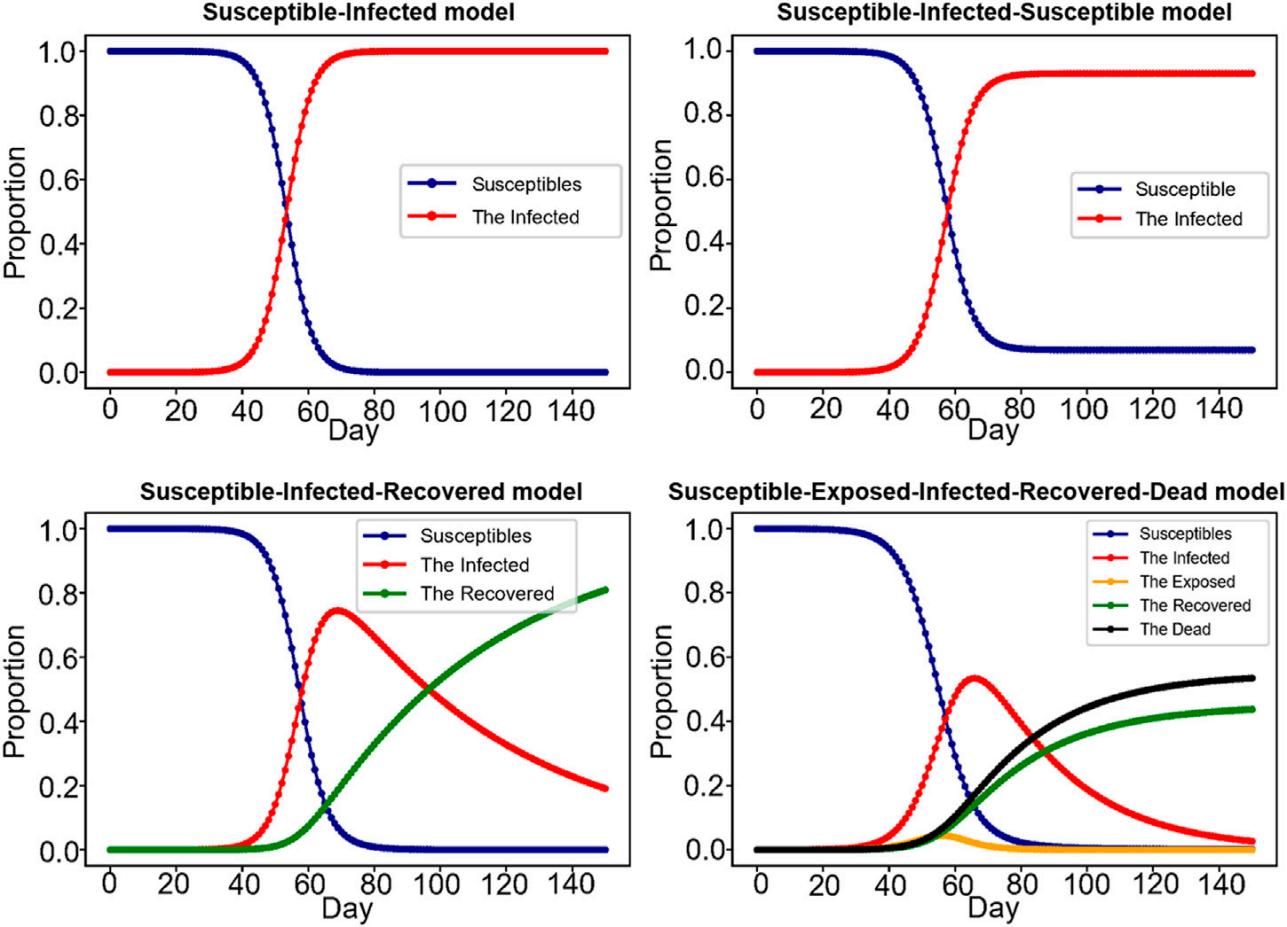
Mathematical model. The picture shows four classic models of infectious diseases, Susceptible-Infected model (SI), Susceptible-Infected- Susceptible model (SIS), Susceptible-Infected-Recovered model (SIR), Susceptible-Exposed-Infected-Recovered-Dead model (SEIRD), with each letter representing a state. For example, SEIRD model represents susceptible, infected, exposed, recovered, and dead.

At present, the most important COVID-19 prediction models at home and abroad mainly focus on traditional mathematical models, such as Susceptible-Infected model (SI), Susceptible-Infected- Susceptible model (SIS), Susceptible-Infected-Recovered model (SIR), Susceptible-Exposed-Infected-Recovered model (SEIR), Susceptible-Infected-Recovered-Dead model (SIRD), Susceptible-Exposed-Infected-Recovered-Dead model (SEIRD), etc., and popular machine learning models (such as Linear Regression model, Polynomial Regression model, Support Vector Machine model, Artificial Neural Network model, etc.). The traditional mathematical model refers to the mathematical analysis of the transmission mode, transmission speed and transmission range of infectious disease on the basis of population number, and expresses it in the form of differential equations. Treating infectious diseases from a mathematical perspective can reveal the internal model and potential structure of epidemic control, and contribute to an in-depth understanding of the transmission dynamics of infectious diseases and the potential effects of different public health intervention strategies (Rahimi et al., 2021). As the World Health Organization puts it, real-time mathematical models play a key role in responding to outbreaks. Supplementary Appendix SA shows some basic mathematical models, their specific differential equations and parameter meanings. Figure 7 shows some basic mathematical models.

Apparently, these are basic mathematical models, and because COVID-19 is subject to so many uncertainties, cultural, economic, political and sociological are critical to understand the epidemic. Therefore, only by taking into account various factors as much as possible, such as seasonal influence, in-and-out rate of national population, infection rate of latent population, ICU beds in hospital, efficiency of receiving and treating, etc. this model can be closer to the actual situation and simulate the real effect. Supplementary Appendix SB shows the current epidemic prediction models and their respective strengths and weaknesses. For example, Wang et al. (Jia et al., 2020) introduced an extended SIR model, which combined real-time isolation measures and expanded the SIR model to adapt to the real-time changing transmission rate in the population, and covered the effects of different epidemic prevention measures. Ivorra et al. (Ivorra et al., 2020) proposed a new *θ*-SEIHRD model (not SIR, SEIR, or other general models) to simulate the propagation of infectious diseases. The model takes into account known characteristics of COVID-19, such as the presence of undetected infectious cases and the different infectious characteristics of hospitalized patients. The method also takes into account the fraction *θ* of detected cases relative to the total number of actual infections, the need for hospital beds can be estimated, and so on. The SEIQR model proposed by Mandal et al. (Mandal et al., 2020). They introduced isolation levels and government interventions, such as lockdown, media coverage of social distancing, and improved public health, to reduce disease transmission. Since many people had little information about the COVID-19 virus in the early stages of the epidemic, Zhao et al. (Zhao et al., 2021) considered that information could influence human behavior, thus influencing the dynamic transmission process of the epidemic layer. Therefore, the proposed SEIR/ V-UA model incorporates an information mechanism to better fit the future development trend of COVID-19.

In addition to mathematical models, the Machine Learning model shown in Supplementary Appendix SC has also become an important tool for researchers (Chimmula and Zhang, 2020; Rustam et al., 2020). One application of Neural Network is for time series prediction algorithm. Neural Network can learn the behavior of time-related data, and can predict the future value. Oliveira et al. (de Oliveira et al., 2021) proposed an Artificial Neural Network model, in which an ANN model was applied to predict the number of confirmed COVID-19 cases and deaths, as well as the time series for the next 7 days in Brazil, Portugal and the United States. Mohimont et al. (Mohimont and Chemchem, 2020) mainly studied a number of models based on CNN, and also proposed a layered transfer learning scheme. Finally, good national and regional accuracy is obtained, and the performance of ordinary CNN is improved. It is now integrated into a COVID-19 surveillance and prediction instrument. Leslie (Leslie and Yeager, 2020) developed a predictive model for the outbreak of COVID-19 in Canada using deep learning (DL) models. The model uses recursive Long Short-Term Memory (LSTM) networks to adapt to the nonlinearity of a given COVID-19 data set, which can overcome the limitations of traditional time series prediction techniques and produce the latest results on time data. Bhimala et al. (Bhimala and Patra, 2021) assessed the relationship between weather factors and COVID-19 cases, and established a predictive model using deep learning model LSTM. The results show that the multivariate LSTM model based on temperature time series data performs well in the high humidity regions of Kerala, Tamil Nadu and West Bengal. It shows that certain high humidity areas are more conducive to the outbreak of COVID-19.

The next, some classical time series prediction models are also favored by researchers. Time series models Autoregressive Integrated Moving Average (ARIMA) and Seasonal Autoregressive Integrated Moving Average (SARIMA) were used to predict COVID-19 pandemic trends in the top 16 countries with 70–80% of the global cumulative cases (ArunKumar et al., 2021). The results showed that SARIMA’s predictions were more realistic than ARIMA’s, confirming the existence of seasonality in COVID-19 data. ARIMA, Brownian exponential smoothing and RNN-LSTM were compared (Guleryuz, 2021). It is found that the ARIMA model can fit the new outbreak situation well. Molin et al. (Molin et al., 2020) compared and analyzed many models, and found that in all scenarios, the models ranked from the best to the worst in accuracy were Support Vector Regression (SVR), Stacking Ensemble Learning, ARIMA, CUBIST, RIDGE, and RF models. Of course, these models have their advantages and disadvantages. Mathematical model with the combination of machine learning model could become a hotspot of research on the future, Zhong team (Yang et al., 2020) considered each province between the flow of population, and use the modified SEIR model and LSTM model, proves the rationality of China’s strict control measures, according to the analysis of China’s the outbreak at its peak in late February 2020, By the end of April 2020, it showed a gradual decline. A 5-day delay would triple the size of the outbreak in mainland China. Lifting quarantine in Hubei will result in a second epidemic peak in Hubei province in mid-March 2020 and extend the epidemic until late April, as confirmed by Machine Learning predictions. The dynamic SEIR model can effectively predict the peak and magnitude of the COVID-19 epidemic. The control measures implemented on 23 January 2020 are essential to reduce the eventual scale of the COVID-19 epidemic.

**FIGURE 8:**
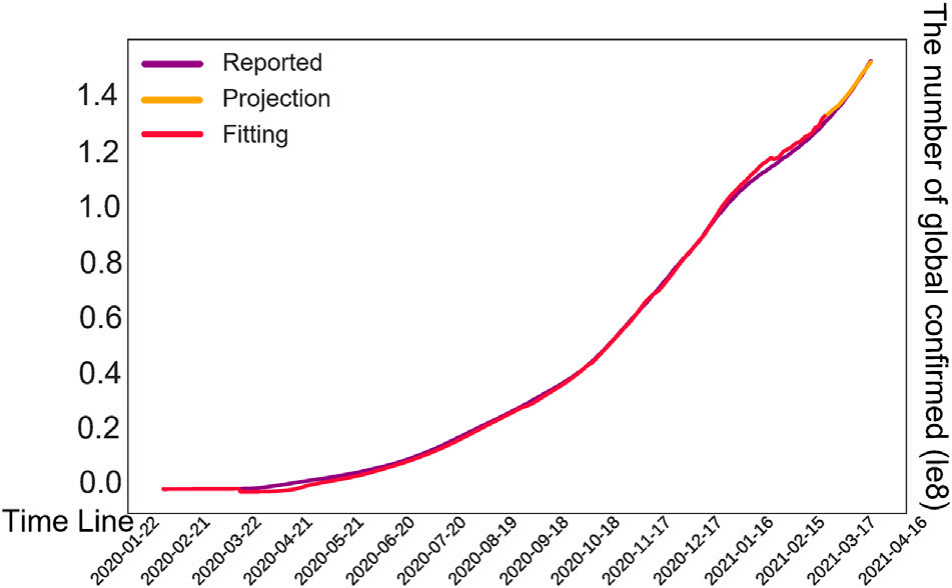
Prediction results of our model. The vertical axis represents the number of global confirmed cases. The red lines are fitted trends, the purple lines are actual cases, and the orange lines are predicted trends over the next month.

**FIGURE 9:**
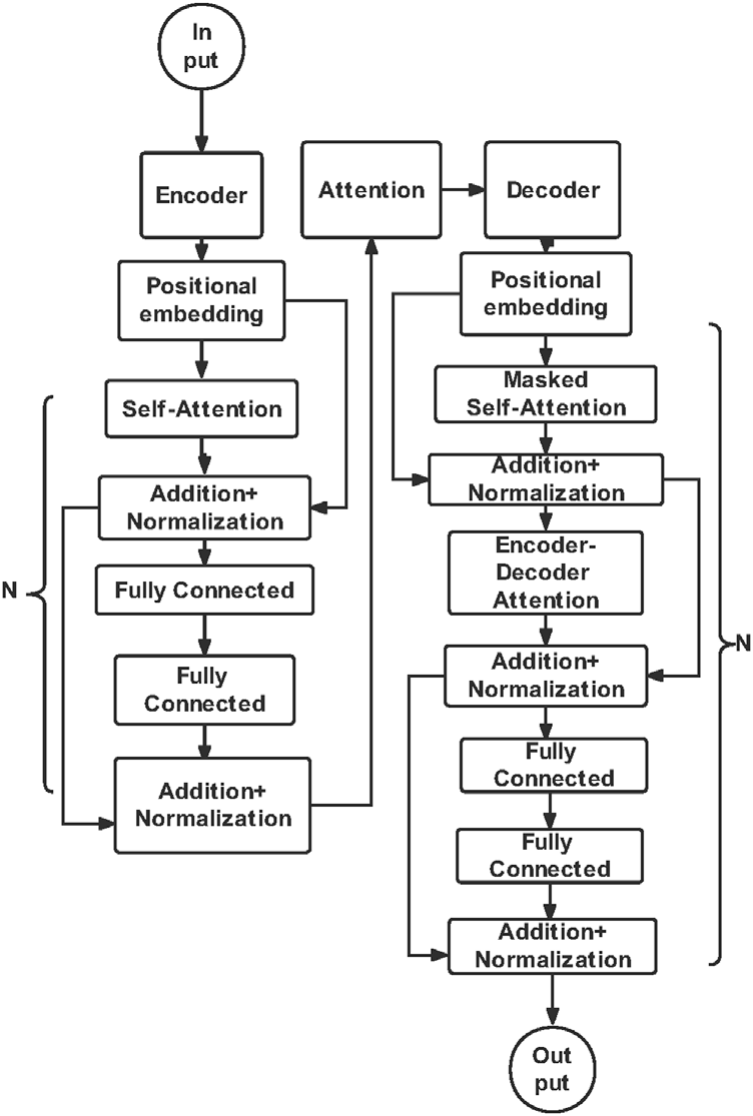
Transformer model structure diagram. Transformer models have Input, Output, Attention mechanisms and Encoder-Decoder architecture. In our T-SIRGAN prediction module, the encoder models the relationships among orders in the sequence, and the decoder learns the variable representation vector.

Finally, we proposed a T-SIRGAN model to predict the future trend of the epidemic (Wang et al., 2022). Due to the lack of data volume, we used Generative Adversarial Networks (GAN) to amplify the data, and replaced the random noise of GAN with the noise regulated by SIR model. Then, Transformers are used to predict the future trends of COVID-19 based on the generated synthetic data. We found that this model performs well compared to LSTM, ARIMA, Decision Tree Regression, SVM, K Neighbors Regression and other models. In addition, the development trend of COVID-19 next month was successfully fitted with an error of 0.0035 MSE, as shown in Figure 8 and Figure 9 shows the model structure of the Transformer model for predictive tasks.

To sum up, the fusion of classical mathematical model of infectious diseases and deep learning model will be a research direction in the future, and the advantages of both can be combined to make a more accurate prediction of the future trend of COVID-19. We also need to study the transmission mechanism of COVID-19 from all angles, including season, temperature, demographic, social, economic, medical, educational and political. Make timely predictions and implement the best control measures to stop the spread of COVID-19.

## Artificial Intelligence Construction of Telemedicine Platform

There are many ways of detecting COVID-19. In addition to nucleic acid tests, clinical manifestations, CT images, etc. In recent years, with the development of 5G technology, telemedicine has gained great development space. We can check our health status with some smart devices. This allows us to see what’s going on in our bodies without leaving the house, and if something goes wrong, we can treat it immediately and prevent it from getting worse. Wosik et al. (Wosik et al., 2020) introduced the role of telemedicine in three stages of American medical service: 1) Home clinics 2) Mitigated the proliferation of pandemic hospitals 3) Post pandemic recovery. The COVID-19 pandemic is forcing all health systems, hospitals and clinics to quickly implement telemedicine services, and telemedicine’s time has come. Due to the large gap between urban and rural medical conditions, Hirko et al. (Hirko et al., 2020) pointed out that the rapid implementation of telemedicine plan in rural areas in response to the COVID-19 pandemic would solve the gap in rural medical conditions to a great extent. In order to build a telemedicine platform, it is necessary to obtain user information in the data system of mobile phone suppliers. Leslie et al. (Leslie and Yeager, 2020) proposed to promote the openness of data so as to promote the construction of telemedicine platform. A full spectrum of researchers will need to be mobilized to understand and respond to the challenges posed by the epidemic.

Apparently, not only the germ of theory, but also Rao et al. (Srinivasa Rao and Vazquez, 2020) proposed a Machine Learning algorithm to collect travel history and common symptoms through online surveys based on smartphones. The data collected can be used to assist in the initial screening and early identification of possible COVID-19 infections. Thousands of data points can be collected and processed through an Artificial Intelligence (AI) framework that can ultimately assess individuals at risk of contracting the virus and categorize them into no risk, lowest risk, medium risk, and high risk. Cases identified in high-risk groups can be isolated earlier, reducing the chance of transmission. Turer et al. (Turer et al., 2020) recommended to use Electronic Personal Protective Equipment (EPPE) to protect employees and preserve Personal Protective Equipment (PPE) during the COVID-19 pandemic, as well as to provide rapid emergency care to low risk patients. Tucker et al. (Tucker, 2020) provided a remote patient monitoring solution for COVID-19 patients (Get Well Loop). Minimizing the exposure rate of COVID-19 patients. Remote patient monitoring is an effective way to manage COVID-19 patients at home.

Telemedicine platforms should provide users with the latest epidemic trends, remind them to take appropriate prevention and control measures, and help users check whether they have had close contact with confirmed cases. If the user has physical discomfort, can immediately call the police or emergency call, so as to get the corresponding isolation and treatment. In the future, the popularization of telemedicine can not only alleviate the shortage of hospital resources during the epidemic, but also monitor the activities of the incubation period population in real time, facilitating screening and controlling the spread of the epidemic. Of course, the premise is to get users’ permission, and protect the security of users’ information, to prevent the use of illegal elements (Islam et al., 2020).

## Discussions and Future Research Directions

Above all, Artificial Intelligence technology plays an extremely important role in the prevention and control of COVID-19, especially in the field of clinical medicine, it can quickly identify the patient’s CT and X-ray images to diagnose the type of pneumonia patients. To learn the clinical data of patients, find out the clinical features of COVID-19 patients, and predict the current severity level, so as to send a warning message to the medical staff. However, this study argues that there are still some challenges regarding the application of Artificial Intelligence algorithms in the field of medicine (Mohamadou et al., 2020).

First of all, the main challenge of COVID-19 detection is the problem of data imbalance. Due to the scarcity of lung image data of COVID-19 patients, the development model cannot be evaluated and tested on a large number of data sets, and the best Artificial Intelligence algorithm cannot be selected. This requires us to establish an open and shared data set for researchers to train and test models (Islam and Islam, 2020). Secondly, there is still a lack of available label data, and extending existing data sets or using a small number of samples in model training are the current strategies that must be chosen. However, most current models are weakly supervised methods, because manual tagging of imaging data is time-consuming and expensive. In the future, we may need unsupervised deep learning models and transfer learning methods to process imaging data. It can not only ensure the accuracy of the algorithm, but also break the limitation of labeled data. Moreover, the diagnosis of medical imaging using artificial intelligence requires sufficient evidence to prove its correctness, because artificial intelligence is regarded as a black box. Thus, the interpretability of artificial intelligence models is of importance in the field of medicine. Finally, medical images cannot fully reflect whether COVID-19 is really infected or not, and a model needs to be established from a multimodal perspective. Artificial Intelligence can learn from Multi-modal clinical data to introduce more intelligence to the medical systems to capture the characteristics of disease, so as to obtain reliable results for COVID-19 diagnosis. To develop a more efficient and versatile system to achieve better clinical medical purposes (Alamo and Daniel, 2020), (Rahman and Sarker, 2021).

In addition, Artificial Intelligence also plays an extremely important role in the discovery of drugs, vaccines, choice of treatment and the medical staff of risk assessment. In the future work, we will not only go toward the direction of more intelligent and precise, but also we need to explore other applications of Artificial Intelligence and modeling for COVID-19 in healthcare (Rahman and Sarker, 2021), (Ricci et al., 2021), (Bhargava and Bansal, 2021). Only in this way, COVID-19 pandemic can be conquered as soon as possible. Finally, we can also use Artificial Intelligence technology to make reasonable predictions of the future development trend of COVID-19, so as to formulate the corresponding prevention and control measures. The British statistician George E.P.Box once said, “All models are wrong, but some models are useful.” The prediction models of the COVID-19 epidemic are also the same, where simple models of the early stages of the growing epidemic can still serve as reference information and provide the basis for more complex transmission models. However, it is not possible to say which model fully matches the spread of COVID-19, so the prediction model is only a way to provide us with early warning, and we should treat it with caution (Ulhaq et al., 2020).

Regarding the development direction of infectious disease dynamics models, this study believes that the combination of mathematical model and machine model learning is the main trend of future development, which can not only improve the adaptability of the model, but also increase the scientific rationality of simulation prediction. Secondly, it is necessary to fully grasp the main factors affecting the development trend of the epidemic, including population migration, seasonal factors, isolation control measures, etc. (Ahmad et al., 2020), and they should be integrated into the model, so as to better fit the spread of the epidemic in reality. Finally, we will explore Multi-modal integration, and future research should incorporate data from other sources, such as social media, mass media.

## Conclusion

One and a half years have passed since the COVID-19 outbreak. During this period of time, vaccines and new treatments have been come out one after another. However, the COVID-19 virus is very cunning. It is constantly mutating in different countries and regions based on local natural geographical environment, population immunity and other factors, seriously threatening human life and health. With the development of Artificial Intelligence technology, more and more researchers are committed to fighting COVID-19 virus through Artificial Intelligence. This paper reviews five aspects of Artificial Intelligence’s the interpretation of medical images, modeling of patient clinical data, finding effective drugs to cure patients, predicting the future development trend of epidemic, and building a remote intelligent medical platform. It also introduces the Artificial Intelligence algorithm used in five major aspects, the data sets used, and evaluates the limitations and advantages of the model. Although the current model has made some achievements, there are still great challenges for the future, especially the openness of data sets and the generalization ability of models. Multimodal models will be one of the main research models in the future. In the end, models just provide some advice and information. The most important thing is to rely on our concerted efforts to protect ourselves, cooperate with the government’s epidemic prevention policies, and actively vaccinate. Only in this way can we defeat COVID-19 at an early date.

## Data Availability

The original contributions presented in the study are included in the article/Supplementary Material, further inquiries can be directed to the corresponding authors.
