# Supplementary material for: A Comprehensive Review of Artificial Intelligence in Prevention and Treatment of COVID-19 Pandemic
Source: Front Genet. 2022 Apr 26;13:845305. doi: 10.3389/fgene.2022.845305 (PMC9086537; doi:10.3389/fgene.2022.845305)
Supplement: Supplementary file 1 [file Table1.pdf]

Appendix A: COVID-19 mathematical prediction model.

Models	Model Structure	Compartments	differential equation
SIR Model	$S \xrightarrow{\beta} I \xrightarrow{\gamma} R$	S:Susceptible<br>I:Infectious<br>R:Recovered+Fatal<br>β: Infectious rate<br>γ: Recovered rate +Fatal rate<br>N:N=S+I+R (total people)	$\frac{d_s}{d_t} = -N^{-1}\beta SI$<br>$\frac{d_I}{d_t} = N^{-1}\beta SI - \gamma I$<br>$\frac{d_R}{d_t} = \gamma I$
SEIR Model	$S \xrightarrow{\beta} E \xrightarrow{a} I \xrightarrow{\gamma} R$	S:Susceptible<br>E: Exposed<br>I:Infectious<br>R:Recovered+Fatal<br>α: The rate of incubation to infection<br>β: Infectious rate<br>γ_1: Incubation recovery rate<br>γ_2: Recovery rate after infection<br>N:N=S+E+I+R (total people)	$\frac{d_s}{d_t} = -N^{-1}\beta SI$<br>$\frac{d_E}{d_t} = N^{-1}\beta SI - (\alpha + \gamma_1) E$<br>$\frac{d_I}{d_t} = \alpha E - \gamma_2 I$<br>$\frac{d_R}{dt} = \gamma_1 E + \gamma_2 I$
SIR-D Model	$S \xrightarrow{\beta} I \xrightarrow{\gamma} R, \quad I \xrightarrow{\sigma} D$	S:Susceptible<br>I:Infectious<br>R:Recovered<br>D:Death<br>β:Infectious rate<br>γ: Recovered rate<br>σ:Fatal rate<br>N=S+I+R+D (total people)	$\frac{d_s}{d_t} = -N^{-1}\beta SI$<br>$\frac{d_I}{d_t} = N^{-1}\beta SI - (\gamma + \sigma)I$<br>$\frac{d_R}{d_t} = \gamma I$<br>$\frac{d_D}{d_t} = \sigma I$

SIR-F Model	$S \overset{\beta}{\rightarrow} S^* \overset{\alpha_1}{\rightarrow} F$<br>$S^* \overset{1-a_1}{\rightarrow} I \overset{\gamma}{\rightarrow} R$<br>$I \overset{\alpha_2}{\rightarrow} F$	S:Susceptible<br>S^*: Susceptible In the incubation<br>I:Infectious<br>R:Recovered<br>F:Fatal<br>α_1: Mortality rates in category S^* population<br>α_2:Mortality rates in category I population<br>β:Infectious rate<br>γ: Recovered rate<br>N=S+I+R+F	$\frac{d_s}{d_t} = -N^{-1}\beta SI$<br>$\frac{d_I}{dt} = N^{-1}(1-\alpha_1)\beta SI - (\gamma+\alpha_2)I$<br>$\frac{d_R}{d_t} = \gamma I$<br>$\frac{d_F}{d_t} = N^{-1}\alpha_1\beta SI + \alpha_2 I$
SEWIR-F Model	$S \overset{\beta_1}{\rightarrow} E \overset{\beta_2}{\rightarrow} w \overset{\beta_3}{\rightarrow} s^* \overset{\alpha_1}{\rightarrow} F$<br>$S^* \overset{1-\alpha_1}{\rightarrow} I \overset{\gamma}{\rightarrow} R$<br>$I \overset{\alpha_2}{\rightarrow} F$	S:Susceptible<br>S^*: Susceptible In the incubation<br>I:Infectious<br>R:Recovered<br>F:Fatal<br>α_1: Mortality rates in category S^* population<br>α_2:Mortality rates in category I population<br>β_1:Infectious rate<br>β_2: Reciprocal of incubation period<br>β_3: Reciprocal of the inverse of the incubation period<br>γ: Recovered rate<br>N= N=S+I+R+F+E+W (total people)	$\frac{d_s}{d_t} = -N^{-1}\beta_1 S(W+I)$<br>$\frac{dE}{d_t} = N^{-1}\beta_1 S(w+I) - \beta_2 E$<br>$\frac{dw}{d_t} = \beta_2 E - \beta_3 W$<br>$\frac{d_I}{dt} = (1-\alpha_1)\beta_3 W - (\gamma+\alpha_2)I$<br>$\frac{d_R}{d_t} = \gamma I$<br>$\frac{dF}{d_t} = \alpha_1\beta_3 W + \alpha_2 I$
