# Supplementary material for: A Comprehensive Review of Artificial Intelligence in Prevention and Treatment of COVID-19 Pandemic
Source: Front Genet. 2022 Apr 26;13:845305. doi: 10.3389/fgene.2022.845305 (PMC9086537; doi:10.3389/fgene.2022.845305)
Supplement: Supplementary file 2 [file Table2.pdf]

Appendix B: Mathematical model of COVID-19.

Model	Countries	Purpose	Result	Advantages	Limitations
CHIME model based on SIR	The Philadelphia	Estimate the timing of a surge in clinical demand and best - and worst-case scenarios for hospital capacity constraints caused by localized COVID-19.	CHIME estimates that demand will exceed existing hospital capacity for 31 to 53 days., the three hospitals will have a total capacity of 3,131 to 12,650 beds, including 338 to 1,608 ICU beds and 118 to 599 ventilators	The model has few parameters, is easy to construct, can be parameterized quickly, and adapt to the characteristics of local population	There was no transition, no consideration for social networks, geographic information, and hospital admissions
Genetic algorithm and SEIR model	Five American states (Georgia, Michigan, New York, Pennsylvania and Virginia)	Predict COVID-19 transmission in the United States and the impact of reopening and utilization of hospital resources	The genetic algorithm is used to match the SEIR model with the existing data	The genome consists of a number of status-related parameters: normal exposure rate, social distancing exposure rate, transmission probability, mortality rate, test eligibility, mask wearing transmission reduction factor and testing period	The SEIR model tends to underestimate the number of cases and overestimate the number of casualties in the early stages of an outbreak, a tendency attributed to potential biases in the data itself, so the model is biased in this way
Modified SEIR model and LSTM	China	Explain how these control measures have affected the spread of the outbreak	The epidemic in China will peak in late February and gradually decline by the end of April. A five-day delay would triple the size of the outbreak in mainland China	Can effectively predict the peak and size of the COVID-19 epidemic. Taking into account the immigration and emigration rates of each province	Other factors that might increase the number of confirmed cases, such as diagnostic capacity, were not considered. Seasonal effects were not considered. Seasonal temperature changes are believed to be an important reason for the dispersal of SARS in Guangdong
Ising model and SIR-D model	India	The SIR (D) model was used to analyze the extent to which this multi-stage blockade was active in "curve flattening" and threat reduction	Quantum computing can be easily used in epidemiological research due to its minimal temporal and spatial complexity. SIRD models suggest that lockdown is highly effective in suppressing infection	Qualitative curves of epidemic and infection propagation are given by quantum circuit design. With the correct use of parameters and some constants, this can be used to simulate the curves of various regions. Add complexity.	Birth and death rates, permanent immunity of recovered patients, inevitable migration and interactions are not taken into account. Make the model lose its precision. No consideration was given to distinguishing exposed individuals from susceptible ones and modeling them accordingly. The trend of recovery and its rate are also difficult to understand due to the lack of demographic data on age, sex, etc., of convalescence-4.Our N(total population) is assumed to be constant, but migration, births and deaths can change N
SIRD model and reciprocal Caputo score	23 countries and regions	A fractional SIRD (susceptibility to infection recovery to death) model based on Caputo derivative is proposed for memory effects (long and short) and	The model was a good predictor of the total number of infections in 23 countries	The proposed Multi-score approach could help predict the total number of infections more effectively so that doctors and healthcare systems can be prepared for different scenarios	Cultural, economic, political and sociological aspects are not taken into account

		experimental time series during an outbreak			
ESIR model and correction factor	Italy and Brazil	Compare the development of COVID-19 in Italy and the total population of provinces with similar populations in China. Prediction of COVID-19 epidemic trends in Italy by revising and calibrating models	In the ESIR model, the average base reproductive number of COVID-19 in Italy and Hunan is estimated to be 4.34 and 3.16, respectively. Under the current state lockdown, there are a total of 182,051 cases of infection (95% confidence interval: 116 114 -- 274 378), ending on 5 August	The model incorporates real-time changing quarantine measures, extends the SIR model to accommodate real-time changing transmission rates in the population, and covers the effects of different quarantine measures at different times	The number of tests is limited, the incubation period is not considered, there are no suspected cases and the number of daily hospitalizations, and some unforeseen factors may affect these estimates
θ-SEIHRD Model	China	Mathematical modeling of the spread of COVID-19 in 2019.It is a new θ-SEIHRD model (not SIR, SEIR or other general models) to simulate the transmission of infectious diseases.	The results show that the control measures are effective and can rapidly reduce the severity of the epidemic. However, the visible impact on society after the controls began was delayed by two weeks. Based on the models and simulations considered in this paper, the basic reproductive number r0 for COVID-19 in China is estimated to be 4.2732	1. The known special characteristics of the disease, such as the presence of undetected infectious cases and the different health and infectious conditions of hospitalized patients, were taken into account.2. The model can also estimate the demand for hospital beds.	1. The population of the country or area under consideration is evenly distributed. Thus, the spatial distribution of epidemics within a territory is not taken into account.<br><br>2. In addition, the current model only applies to countries or areas where the number of people infected with COVID-19 is relevant, where local transmission is the main cause of disease transmission. In this work, transmission between countries was not modeled.<br><br>3. Furthermore, since there is no clear scientific evidence of the effect of humidity and temperature on the Novel Coronavirus, we have not included these two factors in our model.
SEIQR Model	India's Maharashtra, Delhi, Tamil Nadu	Mathematical models can help when it comes to which public health interventions are the best strategies for disease control and how they affect disease dynamics	Our modelling work shows that isolation and government intervention strategies such as lockdowns, media coverage of social distancing and public health can all play an important role in reducing the spread of COVID-19	Isolation levels and government interventions such as lockdowns, media coverage of social distancing and improved public health have been introduced to reduce the spread of the disease	The prediction here is based entirely on some existing parameters. But human behavior is the most uncertain phenomenon. So, if the corresponding parameter space has been altered, there may be some change in the COVID-19 picture
SEIR/V–UA Model	China	The effectiveness of these interventions was tested based on information epidemiological models and data on COVID-19 epidemic cases	To control the spread of infectious diseases, we must strengthen self-protection, improve individual immunity and expand information dissemination	Information can influence behavior change and then influence the dynamic propagation process of epidemic layer.	No comparison with other countries or regions
