# Supplementary material for: A Comprehensive Review of Artificial Intelligence in Prevention and Treatment of COVID-19 Pandemic
Source: Front Genet. 2022 Apr 26;13:845305. doi: 10.3389/fgene.2022.845305 (PMC9086537; doi:10.3389/fgene.2022.845305)
Supplement: Supplementary file 3 [file Table3.pdf]

# Appendix C: COVID-19 machine learning model

| Model | Countries | Purpose | Result | Advantages |
|---|---|---|---|---|
| ANN | Brazil, Portugal and the United States | Artificial neural networks were applied to predict the number of confirmed COVID-19 cases and deaths, as well as the next 7-day time series in Brazil, Portugal and the United States | Neural networks have successfully predicted the number of confirmed cases and deaths from COVID-19.The mean square error of artificial neural network with specific test set is 50% higher than that of artificial neural network with random test set | The combination of the neural network with sigmoid and Liner activation functions and levenberg-Marquardt training functions had the lowest MSE in all cases. This combination has the best performance |
| TCN Model | French | The research focuses on multiple CNN-based (convolutional neural network) models for COVID-19 prediction during the first lockdown in France | The proposed hierarchical transfer learning scheme achieves good country and region accuracy and improves the performance of ordinary CNN.Is now integrated into a COVID-19 surveillance and prediction instrument | The proposed TCN model could achieve a predictive error rate of hospitalization of 1% of confirmed cases at the country level (compared to the proposed CNN of 5%).Good results were also achieved in predicting hospitalization, recovery and death, with error rates of 9 per cent, 3.5 per cent and 4.5 per cent respectively. The proposed transfer learning scheme can improve the learning accuracy of eight regions |
| LSTM Model | Canada, Italy, the United States | Developed predictive models for COVID-19 outbreaks in Canada using state-of-the-art deep learning (DL) models. Assess key features to predict trends and likely cessation times of the current COVID-19 outbreak in Canada and around the world | Based on the results of the LSTM network, we predict that the outbreak could end around June 2020.The data show that the rapid and effective approach taken by Public health authorities in Canada to minimize human exposure has shown a positive impact compared to other countries such as the United States and Italy. Transmission rates in Canada have increased linearly, while in the United States they have increased exponentially. | Recursive LSTM networks, by adapting to the nonlinearity of a given COVID-19 data set, are able to address the limitations of traditional time series prediction techniques and produce up-to-date results on time data. Each block of the LSTM runs in a different time step and passes its output to the next block, until the last LSTM block generates sequential output. |
| LSTM Model | India | The relationship between weather factors and COVID-19 cases was assessed and a predictive model was developed using the deep learning model LSTM | The univariate LSTM model has a good predictive effect on short-term prediction of COVID-19 cases. The LSTM model can be further improved by adding high-resolution weather data, increasing the length of training data and optimizing methods. | Univariate LSTM model is superior to multivariate LSTM model. Univariate LSTM nicely captures trends in the estimation and observation of cases in these states. In high humidity area, multivariate LSTM model based on temperature time series data performs well. |

| | | | | |
|---|---|---|---|---|
| ARIMA and SARIMA Model | The United States, Brazil, South Africa, Chile, Colombia, Bangladesh, India, Mexico, Iran, Peru and Russia | Time series models - ARIMA and SARIMA - are used to predict COVID-19 pandemic trends in the top 16 countries with 70-80% of the global cumulative cases | The ARIMA model's predictions are more realistic than those of the ARIMA model, confirming the presence of seasonality in COVID-19 data, and the results of the study not only shed light on the future trends of the COVID-19 epidemic in the top 16 countries, but also guide the development of health care policies in these countries for the ongoing pandemic | ARIMA and SARIMA models had roughly the same predicted COVID-19 values on day 60, but SARIMA was superior to ARIMA in capturing seasonality or trends in the data. SARIMA's forecast is a more realistic number, as it takes into account the changes that have taken place in the COVID-19 time series over the past few weeks (June-July 2020) and projects into the future |
| SVR, stacking-ensemble learning, ARIMA, CUBIST, RIDGE, and RF models | Brazilian states with high daily incidence include Amazonas, Bahia, Ceara, Minas Gerais, Parana, Rio de Janeiro, Rio Grande Do Norte, Rio Grande Do Sul, Santa Catarina and Sao Paulo | In the superposition ensemble learning method, cubic regression, RF, RIDGE and SVR models are used as the basic learners, and Gaussian process (GP) is used as the meta-learners. The validity of the model was evaluated based on improved index, mean absolute error and symmetric mean absolute percentage error criteria | In all scenarios, the models ranked, from the best to the worst in accuracy, are THE SVR, Stacking -ensemble Learning, ARIMA, CUBIST, RIDGE, and RF models. | Once SVR and overlay integrated learning models are able to learn the inherent nonlinearity of the assessed epidemiological time series, they can be adapted to predict the majority of COVID-19 cases in adoption states. |
| ARIMA, Brownian exponential smoothing and NN-LSTM | Turkey | Build a prediction model that takes into account Turkish statistics. Box-jenkins method (ARIMA), Brown exponential smoothing model and RNN-LSTM are used. | The predicted value of the number of indicators is stable over time. The number of cases in Turkey is not expected to increase in the near future. In addition, from 17 to 31 May, the pandemic will stabilize and mortality rates are not expected to increase. | ARIMA models can be used in new outbreak situations to ensure health and safety .Rapid and accurate development of epidemic prevention measures is key to epidemic prevention and management, so corrective and preventive measures can be updated based on the available data. |
| LR、LASSO、SVM、ES Model | Australia, Canada, Algeria | Four standard prediction models, including linear regression (LR), minimum Absolute contraction selection operator (LASSO), support vector machine (SVM) and exponential smoothing (ES), were used to predict COVID-19, such as the number of new infections, the number of deaths, and the number of people recovering in the next 10 days. | Applying these approaches to the current COVID-19 pandemic scenario is a promising mechanism. It has also helped authorities to take timely action and decisions to contain the COVID-19 crisis. | ES performed best of all models, LR and LASSO performed best at predicting newly confirmed cases, mortality and cure rates, while SVM performed poorly across all predicted scenarios for a given data set. |
